# Diagnosis of left ventricular hypertrophy using convolutional neural network

**DOI:** 10.1186/s12911-020-01255-2

**Published:** 2020-09-25

**Authors:** Zini Jian, Xianpei Wang, Jingzhe Zhang, Xinyu Wang, Youbin Deng

**Affiliations:** 1 Electronic Information School, Wuhan University, Wuhan, P.R. China; 2 Department of Medical Ultrasound, Tongji Hospital, Tongji Medical College, Huazhong University of Science and Technology, Wuhan, P.R. China

**Keywords:** Echocardiography, Deep learning, Diagnosis of left ventricular hypertrophy, Convolutional neural network

## Abstract

**Background:**

Clinically, doctors obtain the left ventricular posterior wall thickness (LVPWT) mainly by observing ultrasonic echocardiographic video stream to capture a single frame of images with diagnostic significance, and then mark two key points on both sides of the posterior wall of the left ventricle with their own experience for computer measurement. In the actual measurement, the doctor’s selection point is subjective, and difficult to accurately locate the edge, which will bring errors to the measurement results.

**Methods:**

In this paper, a convolutional neural network model of left ventricular posterior wall positioning was built under the TensorFlow framework, and the target region images were obtained after the positioning results were processed by non-local mean filtering and opening operation. Then the edge detection algorithm based on threshold segmentation is used. After the contour was extracted by adjusting the segmentation threshold through prior analysis and the OTSU algorithm, the design algorithm completed the computer selection point measurement of the thickness of the posterior wall of the left ventricle.

**Results:**

The proposed method can effectively extract the left ventricular posterior wall contour and measure its thickness. The experimental results show that the relative error between the measurement result and the hospital measurement value is less than 15%, which is less than 20% of the acceptable repeatability error in clinical practice.

**Conclusions:**

Therefore, the measurement method proposed in this paper has the advantages of less manual intervention, and the processing method is reasonable and has practical value.

## Background

Left ventricular hypertrophy (LVH) is in the heart of the left ventricular myocardial morphology changes, increase in number, resulting in left ventricular wall thickening [1]. Left Ventricular Posterior Wall Thickness (LVPWT) will be significantly thickened in patients with LVH [2]. LVH is generally considered to be potentially associated with heart failure, arrhythmia and other diseases [3]. Left ventricular hypertrophy is considered as a reliable index in the diagnosis of organic heart disease, and it is of great significance to evaluate the disease development and prognosis. Therefore, early and accurate diagnosis of the disease can provide reliable reference for follow-up treatment and reduce the probability of risk events. The screening basis of left ventricular hypertrophy in modern medicine is based on electrocardiogram and echocardiography. Sometimes accurate measurements are confirmed by cardiovascular magnetic resonance imaging (CMR) [4]. Echocardiography can measure the degree of hypertrophy between the ventricular wall and the muscle, which is more intuitive, accurate and more sensitive than the electrocardiogram [5]. Studies have shown that the sensitivity of using Doppler echocardiography to diagnose left ventricular hypertrophy is up to 88% ~ 100% [6].

Common methods used in medical image analysis include edge detection, texture feature extraction, shape modeling and template matching [7], etc. At present, the research directions of deep learning in medical images mainly include medical image classification and medical target recognition, localization and detection of lesions, and medical image segmentation of tissues and organs. The great progress and excellent performance of deep learning in the field of computer machine vision has promoted its application in medical image analysis, but it has not been widely used in echocardiography. Echocardiography is a uniquely well-suited approach for the application of deep learning in cardiology [8]. Deep learning can be applied to medical image processing, and the position of lesions can be detected automatically by computer, and the degree of lesions can be judged and estimated, which can not only improve the detection accuracy, but also greatly reduce the workload of doctors.

In recent years, the application of image classification technology in medical imaging has developed rapidly. CNN performs well in the diagnosis of many diseases based on image classification, but there are few data on how to apply CNN to the diagnosis and prediction of heart diseases. Alvaro [9] et al. constructed a time-distribution convolutional neural network with long-term memory using AdaGrad27 and studied time series data from 600 patients using RMSProp26 algorithm. They found that the ability of deep neural network to predict one-year survival rate on cardiac imaging results was better than that of trained cardiologists. Ali [10] et al. developed a deep learning classifier for the prediction task of cardiology, used the pipeline supervision model to focus on relevant structures, trained the classifier with the training set and test set of more than 200,000 images, and completed the effective classification of the image view of still echocardiography. Then, a semi-supervised generative antagonistic network was developed to learn from marked and unmarked data in a general way on the premise that only 4% of the marked data was used in the monitoring technology alone, which achieved high classification accuracy in view classification. On the basis of full convolution neural network, Kai Zhu et al. [11] added the method of key points location and image convex hull to segment the ventricles of echocardiography of the heart, with excellent results.

All the work showed that CNN did a good job in the diagnosis of diseases, including LVH, but it only judged whether the patient was sick or not, without giving the thickness of the left ventricular posterior wall. Although convolutional neural network classifier can screen patients with left ventricular hypertrophy with high accuracy, it cannot obtain specific evaluation indexes of left ventricular hypertrophy. Being able to determine the extent and extent of left ventricular hypertrophy is also of great help in the development of treatment plans and prognosis estimation [12]. Many LVH patients also have other wall hypertrophy, such as ventricular septum, but in this paper, the left ventricular posterior wall thickness was used as the measurement index. Therefore, this paper analyzes the basic components of neural network, neurons and simple neural network, designs the model of convolutional neural network, screens, classifies, cuts and marks the obtained sample images, and trains the network model accordingly. In addition, the algorithm was designed to enhance the image of the target region of the left ventricle on the premise of retaining the detailed features of the edge of echocardiography, so as to effectively extract the contour of the back wall of the left ventricle and measure its thickness.

## Methods

### Detection and image enhancement of target region of left ventricle based on CNN

**Fig. 1:**
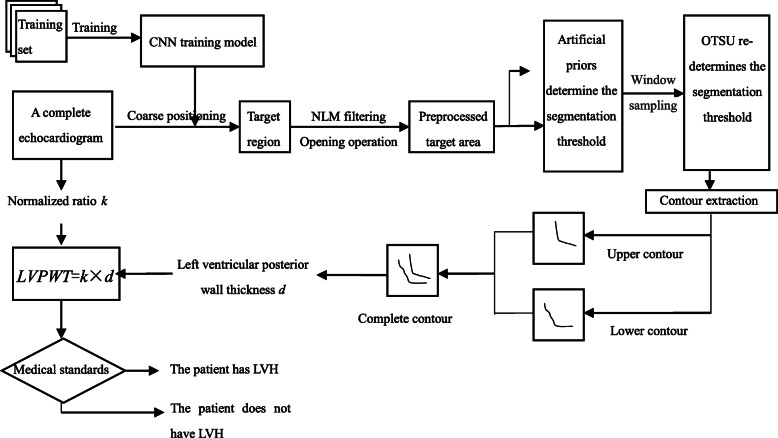
Complete processing flow chart

The entire data set in an echocardiogram is large, and the image of the non-target region will affect the image processing effect. In order to improve the efficiency and effect of image enhancement, this paper firstly uses convolutional neural network for coarse positioning of ultrasonic images, and then carries out image processing calculation for the target region. The specific treatment procedure was as follows: CNN was used to coarse positioning the target region of the left ventricular posterior wall of complete echocardiography. Non-local Mean Filtering (NLM) and opening operation are used to enhance the image of the target region. Then the image of the posterior wall of the left ventricle was segmented based on the prior threshold value of OTSU algorithm, and the upper and lower contour of the posterior wall were extracted respectively. The distance between the upper and lower contours was normalized to obtain the final measurement result of LVPWT, and then to determine whether the patient had left ventricular hypertrophy. The entire processing process is shown in Fig. 1.

### CNN model design

**Fig. 2:**
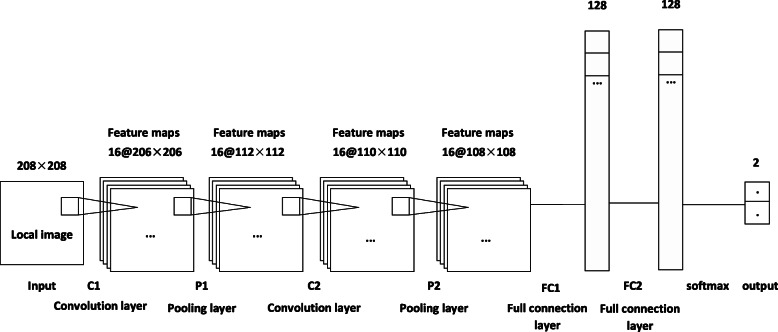
CNN structure

In 1962, Hubel and Wiesel [13] found that some nerve cells in the brain can only respond to the edge in a specific direction, that is, some of the neurons are only excited to the vertical edge, and some are only excited to the horizontal or diagonal edge. Hubel and Wiesel found that the neurons were all arranged in columnar structures, and that visual perception could only be produced when all the neurons worked together. The idea that specific components in neurons have specific tasks (neurons in the visual cortex look for specific characteristics) is also applicable to the field of deep learning [14]. In this paper, the network model used for the location of left ventricular target area is shown in Fig. 2, which consists of 7 layers, including 2 layers of convolution layer (C1, C2), 2 layers of pooling layer (P1, P2), 2 layers of full connection layer (FC1, FC2) and 1 layer of softmax regression layer. The C1 layer is composed of 16 feature maps of 206 × 206. The neurons of each feature graph are connected to the input layer. The size of the convolution kernel is 3 × 3 and the step length is 1. The input image with the size 208 × 208 was traversed, resulting in 16 different 206 × 206 feature maps. The P1 layer is a pooling layer. The convolution layer is sampled by using CNN’s local connection characteristics, which can reduce the amount of processed data and speed up the convergence time while retaining useful information. In the pooling layer, the size of convolution kernel is 3 × 3 and the step length are 2. After processing, 16 feature maps of 112 × 112 are obtained. The C2 layer is also a convolutional layer. The size of the convolution kernel is 3 × 3, and the step size is 1, and 16 feature maps of 110 × 110 are obtained. The convolution kernel size in the P2 layer is 3 × 3, and the step size is 1, and 16 feature maps of 108 × 108 are obtained. FC1 layer is a fully connected layer connected with P2, and FC2 layer is a fully connected layer connected with FC1. The number of FC1 layer and FC2 layer is 128. After the full connection layer FC1 and FC2, the Dropout layer is added. During the CNN training process, each sample randomly discards the input and output of some neurons with probability P = 0.5 during each iteration, reducing the complex co-adaptability between neurons to reduce over fitting [15]. For this paper, there are only two types of samples to be tested (target area and non-target area), so the softmax regression layer has two outputs.

The convolutional neural network can extract features directly from the image. In this paper, a 200 × 200 sliding window is set, which takes a step size of 25 and traverses the entire echocardiogram to achieve the detection of the target area. The image of the posterior wall of the left ventricle has obvious position feature and gray value feature. In order to improve the detection efficiency, region selection and gray value selection are carried out before convolution neural network is used for probability prediction. Set the threshold value of the average gray value of the window. If the threshold value is lower than this threshold value, no probability prediction will be made, and the window will be filtered directly.

### Target area image enhancement

Ultrasound image processing of the heart is difficult, on the one hand, due to the continuous beating of the heart and the complexity of the structure, on the other hand, the quality of the ultrasound image is poor relative to other medical imaging systems. Scattering occurs when ultrasound interacts with human tissue targets of comparable wavelengths. The scattered echo overlaps and interferes with each other, which will produce the common speckle noise in the ultrasonic image, which is shown as irregular spots in the image [16]. Excessive speckle noise will destroy the quality of ultrasound image and affect the results of artificial analysis and computer-aided diagnosis. Therefore, it is necessary to use appropriate algorithms to preprocess the obtained image and keep the details of the image. In this paper, the image preprocessing before the measurement of left ventricular posterior wall is mainly nonlocal means filtering and opening operation.

### Non-local means filtering

Non-local mean filtering is a cooperative filtering method proposed by Buades A. et al. based on SUSAN (Small Univalue Segment Assimilating Nucleus) filtering method, which has been applied to denoising of some medical images, such as MRI, ultrasound imaging, etc. The effect is better than the existing diffusion or wavelet method [17].

Non-local means filtering can be regarded as a special case of local mean filtering. The principle is that in addition to the filtering window, a search box is set to find pixels similar to the center pixel of the current filtering window. When estimating the gray value of the central pixel, the pixels with similar central pixel structure are given larger weight values, and the output value of the central pixel is obtained after weighted averaging [18]. The non-local mean filtering result *y*(*i*) principle expression is as follows:

1 $$ y(i)=\sum \limits_{j\in X}w\left(i,j\right)x(j) $$

Where *x*(*j*) is the image noise sub-block, and ${\sum}_{j\in X}w\left(i,j\right)$ is the similarity index between pixels *i* and *j*. The calculation formula is as follows [19, 20]:

2 $$ \sum \limits_{j\in X}w\left(i,j\right)=\frac{1}{Z(i)}{e}^{\frac{-\sum {\left\Vert x(i)-x(j)\right\Vert}_a^2}{h^2}} $$

Where *w*(*i*, *j*) ∈ [0, 1], *a* is the standard deviation of Gaussian kernel, *h* is the filtering intensity, $\sum {\left\Vert x(i)-x(j)\right\Vert}_a^2$ is the weighted Euclidean distance, which is negatively correlated with the similarity degree of the two image blocks. *Z*(*i*) is the normalization coefficient, which is calculated as follows [19, 20]:

3 $$ Z(i)=\sum \limits_j{e}^{\frac{-\sum {\left\Vert x(i)-x(j)\right\Vert}_a^2}{h^2}} $$

The non-local mean algorithm takes into account the local similarity between pixels and neighboring image blocks during the processing of each pixel of the image [21]. The information contained in the regional image block can better reflect the detailed characteristics of the image than the information of a single pixel. Therefore, NLM filtering can consider the overall similarity between pixels, and maintain the detailed information of the image while denoising.

### Opening operation

**Fig. 3:**
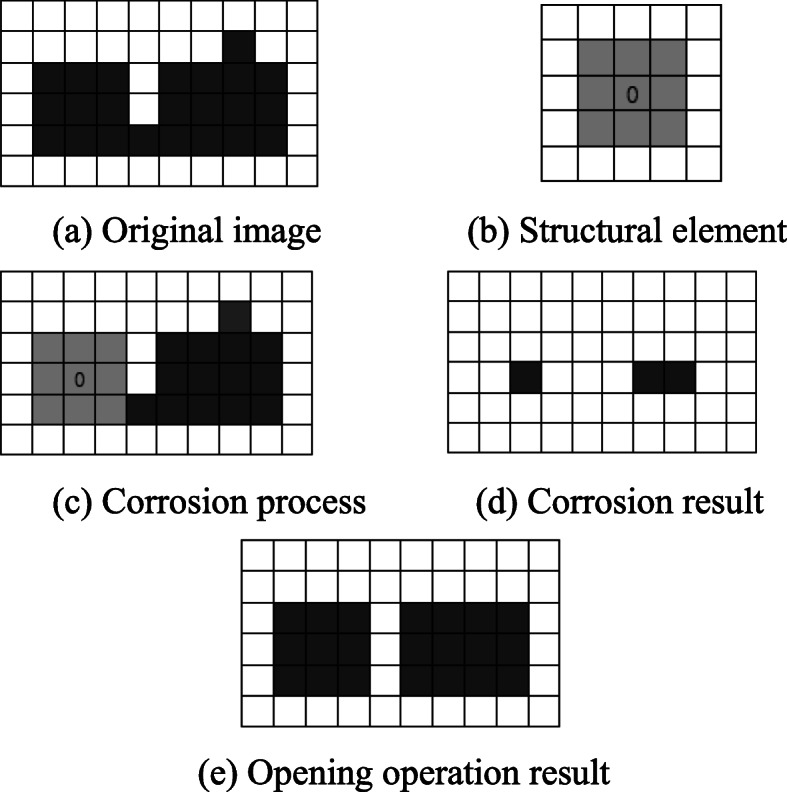
Opening operation schematic. **a** Original image. **b** Structural element. **c** Corrosion process. **d** Corrosion result. **e** Opening operation result

The opening operation is a filter based on geometric operation, including corrosion and expansion operation. The opening operation is equivalent to performing internal low-pass smooth filtering on the processed image, and can remove isolated glitches and small bridges on the premise of keeping the overall shape of the image unchanged. The processing of the opening operation is shown in Fig. 3. Figure 3a is the original image, the white part represents the background, and the gray part represents the target. Figure 3b is the structural element, and the origin position is marked with 0. Figure 3c is the corrosion process, and the image is traversed. Figure 3d is the corrosion result. Figure 3e shows the Opening operation result after the expansion operation.

### Left ventricular hypertrophy diagnosis based on image processing

#### Left ventricular posterior wall contour extraction

After the non-local average filtering and opening operation processing of the target area, the image quality has been significantly improved. To accurately measure the thickness of the posterior wall of the left ventricle, it is necessary to effectively extract the complete contour of the posterior wall of the left ventricle. There are many commonly used edges detection operators in image processing such as: Canny, Sobel, Prewitt, etc. However, the results of their edge detection are usually discontinuous, the complete left ventricular posterior wall contour cannot be extracted, and subsequent thickness measurement is difficult. Therefore, this paper designs an edge detection algorithm based on threshold segmentation to achieve the effective extraction of the complete contour of the left ventricular posterior wall.

Threshold segmentation is a region-based image segmentation technology, which can connect regions with different gray values. It is suitable for images with different gray levels between target and background. Threshold segmentation compresses the processing volume of data and can improve the processing speed. However, in order to achieve good segmentation effect, it is necessary to determine a reasonable segmentation threshold. In this paper, the determination of segmentation threshold is combined with the manual empirical selection method and the OTSU method. The specific scheme is to determine the gray value range of the left ventricular posterior wall contour through a priori analysis of the target region. The maximum and minimum values in this range were selected as the first gray threshold to segment the upper and lower contours of the posterior wall of the left ventricle respectively. A sampling window was automatically selected near the contour edge of the segmentation of the posterior wall of the left ventricle, and then the threshold was adjusted twice after OTSU binarization to improve the segmentation accuracy.

OTSU method is a global binary algorithm, which uses different gray features of the target and background in the image for segmentation [19]. When taking the optimal threshold, the difference between the two parts should be the largest, and the variance between the corresponding two parts should be the largest and the probability of misclassification should be the smallest. T is denoted as the segmentation threshold, and the proportion of the number of pixels of the target and background in the image is denoted as *ω*_0_, *ω*_1_, respectively. The average gray scale is *μ*_0_ and *μ*_1_. The average gray scale of the image is denoted as *μ*, and the variance *g* of the foreground and background images is calculated as follows:

4 $$ u={\omega}_0\times {u}_0+{\omega}_1\times {u}_1 $$

5 $$ g={\omega}_0\times {\left({u}_0-u\right)}^2+{\omega}_1\times {\left({u}_1-u\right)}^2 $$

The above formula can be obtained:

6 $$ g={\omega}_0\times {w}_1\times {\left({u}_0-{u}_1\right)}^2 $$

When the gray-scale segmentation threshold is T^′^, the variance *g* is the largest, and T^′^ is the optimal threshold. It is considered that the target is better segmented from the background at this time.

### Calculation of left ventricular posterior wall thickness

At present, in clinical practice, LVH is a subjective assessment, in which doctors usually evaluate the character and function of the heart by observing several videos within a few minutes and give medical results [22, 23]. The calculation formula of left ventricular posterior wall thickness can be expressed as:

7 $$ LVPWT=k\times d $$

Where k is the normalized scale, d is the distance between the two edge pixels of the posterior wall, and LVPWT is calculated in mm.

The distance between the pixels of the left ventricular posterior wall and the lower edge of the left ventricular posterior wall is usually smooth. Therefore, the distance between the pixels of the two edges of the posterior wall is measured by selecting points in the lower contour. The method of selecting points is as follows: The intersection point between the lower edge contour and the line group with slopes of 45° at different intercepts is selected as the measurement point of the lower edge. Due to the distribution of pixels, for the distribution of pixel points in the neighborhood of 2 × 2, there is no intersection between the 1–3 distribution of pixels and the line, so the selection points in this case need to be filtered out. By setting several sets of different intercepts, the set of measurement selection points of the lower edge is obtained. Search for the point with the minimum distance from the coordinate selection point of all pixel points on the upper edge and take this distance as the reference point. The reference distance group was obtained. After removing the maximum and minimum values of the distance in the distance group, the mean value was taken as the calculated value *d* of the pixel distance between the two edges of the posterior wall of the left ventricle.

In order to obtain better observation results, doctors will appropriately adjust the proportion of images when performing cardiac ultrasound examinations on different patients to meet the needs of diagnosis. To calculate the thickness of the posterior wall of the left ventricle, the scale of the ultrasound image needs to be determined. Compared with the echocardiographic image, the scale is smaller in size, only a few pixels in length, and the position of the scale of different samples is different, which is difficult to cut through simple images or transform the gray value by linear programming to remove the fan-shaped area.

During the processing, a low gray value is first taken for binarization, and a large number of non-target low gray value segments are reduced while the gray value of the scale segment is increased. Then by selecting an appropriate length threshold, the line segments outside the range of the scale line segment length are screened out, and a smaller area threshold is set. And adjust the gray value of all pixels in the outline to 255, remove the fan-shaped area on the right, and generate the remaining line segments on the newly created mask, which is the reference scale line segment. By calculating the length of the scale line segment, the normalized scale of the echocardiogram can be obtained.

The normalized scale calculation formula is:

8 $$ k=\frac{l}{d}\times 10 $$

Where *l* is the pixel distance corresponding to the unit length of the scale line segment, and *d* is the pixel distance between the two edges of the posterior wall of the left ventricle. Then according to eq. 7, the machine measurement value of the thickness of the posterior wall of the left ventricle of the echocardiography can be calculated, and the diagnosis of LVH can be made by referring to relevant clinical standards.

## Results

### Data set acquisition and training

The echocardiographic data used in the experiments in this paper were taken from Tongji Hospital affiliated to Tongji medical college of Huazhong University of Science and Technology. A total of 50 echocardiographic videos of 50 examiners were selected, and different frames of a cardiac cycle were intercepted to obtain more than 2000 echocardiograms. In order to ensure the quality of training, the echocardiograms of each examiner were screened and tailored, and high-quality images of the same plane at the same time of the cardiac cycle were selected, and finally 300 images of the target area and non-target area of the posterior wall of the left ventricle were obtained respectively, each with a size of 200 × 200. Before the training of the convolutional neural network with these 600 images as the training set, the size of all the images was adjusted to 208 × 208, and part of the training sample patterns are shown in Table 1.

**Table 1:**
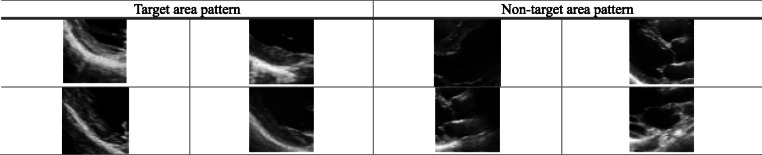
Partial training sample pattern

The development tool of this paper is Pycharm and the learning framework is tensorflow-gpu. During the training of the CNN model, the number of iterations was set as 5000, Batch Size = 20, and the learning rate was 0.0001. In this paper, when training the convolutional neural network, the ReLU function is used as the excitation function to randomly initialize the convolutional layer and the full connection layer, and only the neurons in the activated state are convoluted.

### CNN positioning results

**Fig. 4:**
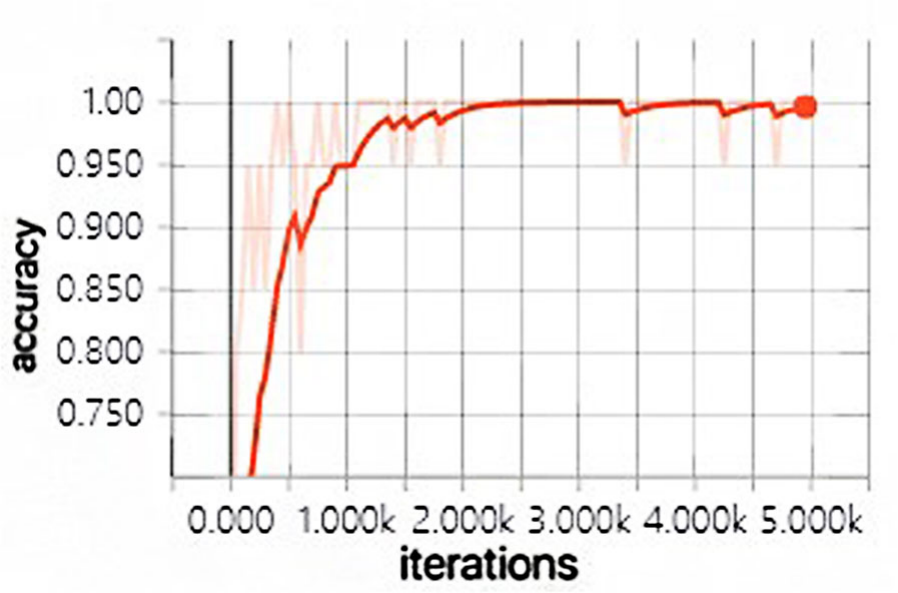
Accuracy change chart

**Fig. 5:**
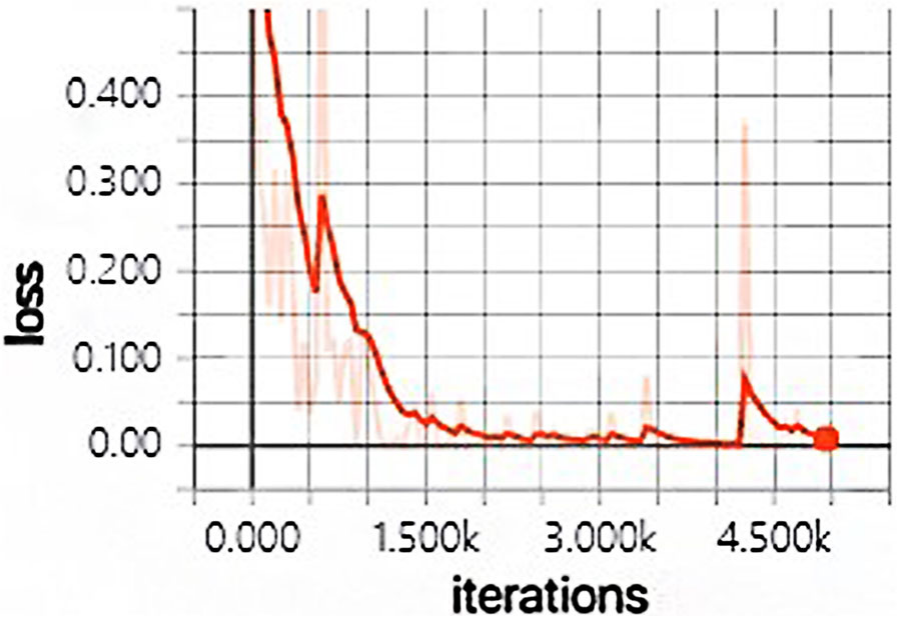
Loss change chart

The model training takes 49 min and 32 s in total, and the changes of the accuracy and loss in the whole training process are shown in Fig. 4 and Fig. 5 below. The light colored lines represent the training set, and the dark lines represent the verification set.

**Fig. 6:**
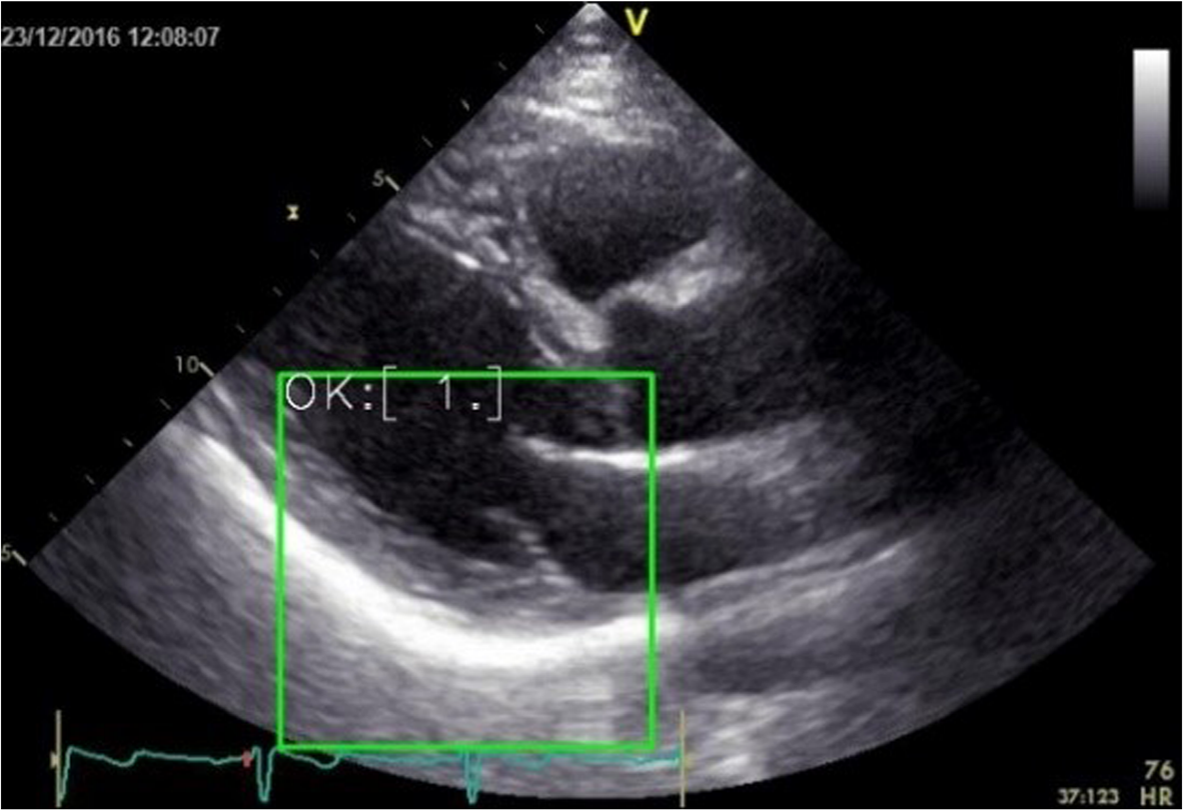
An echocardiogram based on CNN positioning results

In this paper, 10 complete echocardiograms with good quality and diagnostic significance are selected. The trained model was used to test the positioning effect of the target area. The results show that the detection rate of the target area using the convolutional neural network is very high, and the detection effect of a certain echocardiogram is shown in Fig. 6 below. In the frame of the Fig. 6 is the result of target region location based on convolution neural network.

The processing results of 10 complete echocardiograms are shown in Table 2 below. The probabilistic prediction time required for each image with a size of 200 × 200 is approximately 0.6 s. It can be seen from Table 2 that the effective positioning rate in this paper is relatively high. However, there are differences in the number of potential windows filtered by different samples, resulting in different detection time of different samples. Due to the different sample acquisition time, doctors have different image grayscale Settings for medical equipment. In order to ensure the detection efficiency, this paper sets different average gray value thresholds during the screening of different sample windows in the sample test, in which the gray value threshold of sample 1–8 is 50, and the gray value threshold of sample 9–10 is 70.

**Table 2:** The results of each sample based on convolutional neural networks

Sample no.	Number of potential Windows after filtering	Coarse positioning time /s	Effective positioning(Y/N)
No.1	18	11.04	Y
No.2	52	30.02	Y
No.3	43	26.86	Y
No.4	63	38.00	Y
No.5	56	31.64	Y
No.6	29	20.92	Y
No.7	26	15.25	Y
No.8	64	41.03	Y
No.9	94	52.66	Y
No.10	49	32.00	Y

### Target area image enhancement

Studies have shown that speckle noise will form local image features in the flat region of the image, and strong filtering is required to eliminate them [17]. NLM filtering has a good effect on the processing of low-noise images, but echocardiography has dense speckle noise. If the filtering intensity *h* is increased for better denoising effect, the left ventricular contour and other features of echocardiography will be seriously damaged. After test and comparison, during NLM filtering, the window size was set as 7 and the search window size was 21. In order to reduce the damage degree of left ventricular contour details, the filtering intensity *h* was set as 8.

**Fig. 7:**
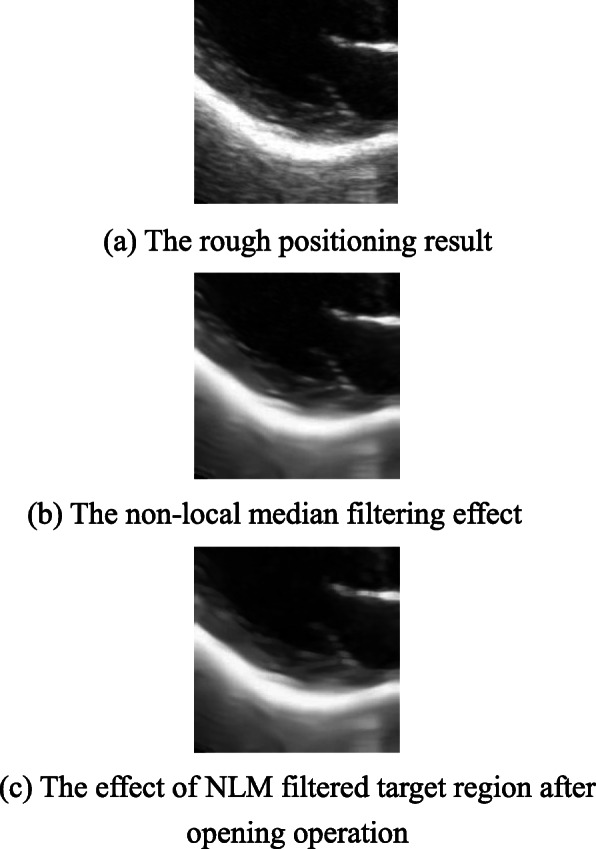
Image enhancement effect map. **a** The rough positioning result. **b** The non-local median filtering effect. **c** The effect of NLM filtered target region after opening operation

The rough positioning result of an echocardiography sample is shown in Fig. 7a, the non-local median filtering effect of the corresponding target region is shown in Fig. 7b, and the effect of the target region filtered by NLM after opening operation is shown in Fig. 7c. It can be seen that after the non-local mean filtering and opening operation processing, the image quality of the target area has been significantly improved. On the premise of preserving the edge contour details, the image quality has been effectively denoised.

### Contour extraction effect

According to the algorithm designed in the scheme, an edge detection algorithm based on threshold segmentation was applied to the preprocessed image of the target region of the left ventricle of a sample. The effect diagram of each stage after the algorithm processing of the upper and lower contour is shown in Table 3.

**Table 3:**
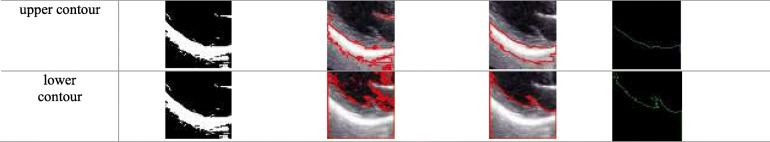
A sample outline extraction stage renderings

**Fig. 8:**
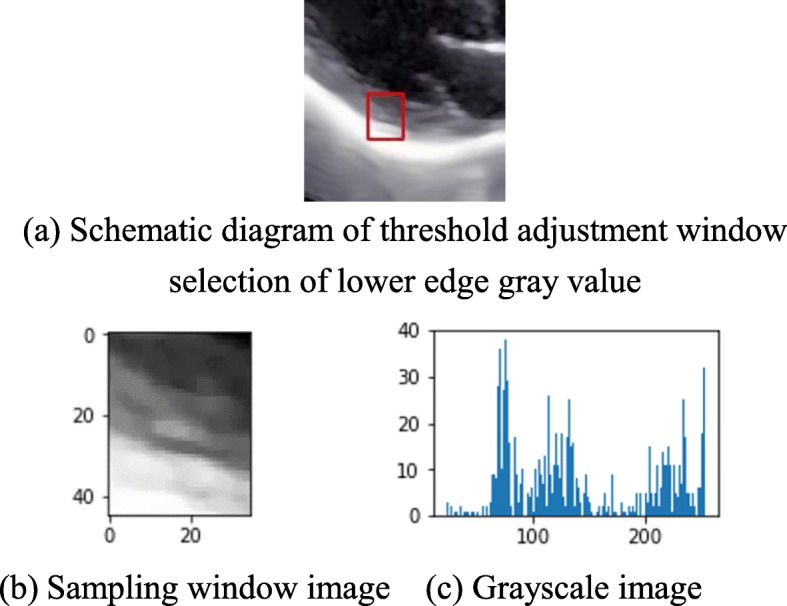
A sample threshold segmentation sampling window. **a** Schematic diagram of threshold adjustment window selection of lower edge gray value. **b** Sampling window image **c** Grayscale image

**Fig. 9:**
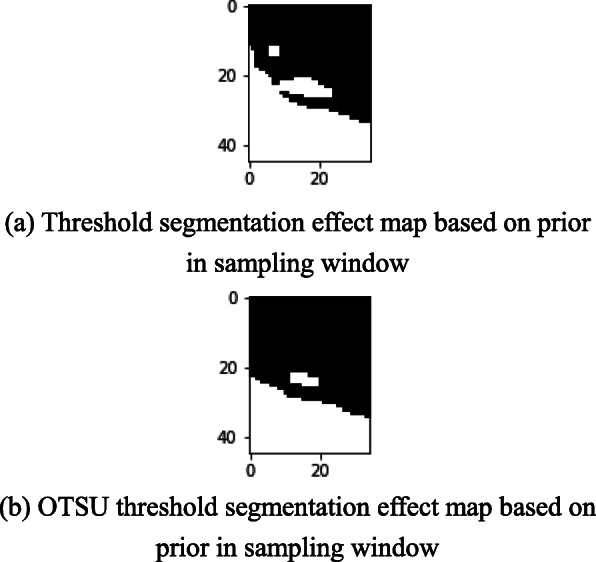
Comparison of prior analysis and OTSU effects. **a** Threshold segmentation effect map based on prior in sampling window. **b** OTSU threshold segmentation effect map based on prior in sampling window

**Fig. 10:**
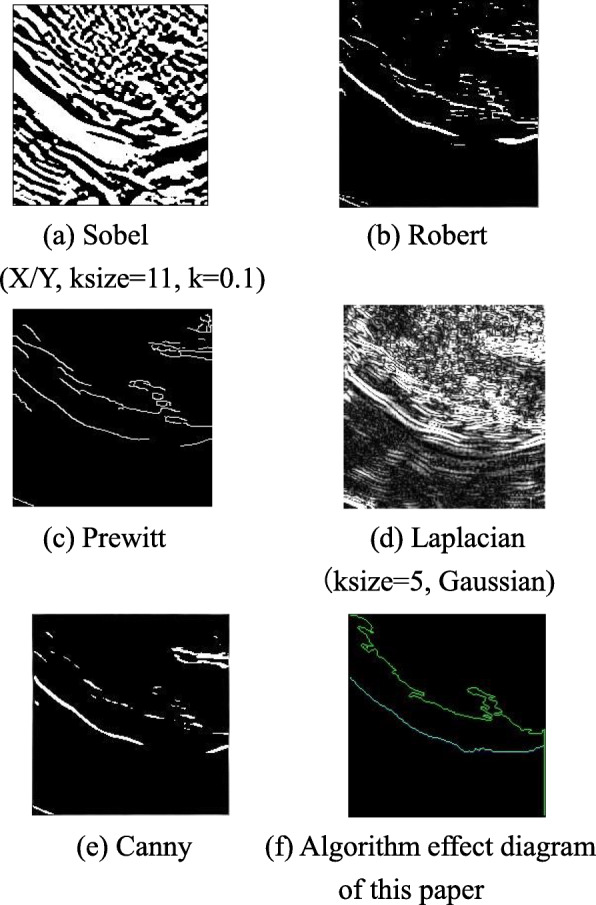
Comparison of processing effects of each algorithm. **a** Sobel (X/Y, ksize = 11, k = 0.1). **b** Robert. **c** Prewitt. **d** Laplacian (ksize = 5, Gaussian). **e** Canny. **f** Algorithm effect diagram of this paper

OTSU binarization is applied to the selected sampling window to readjust the gray value threshold of threshold segmentation. The Fig. 8a shows the schematic diagram of threshold adjustment window selection of lower edge gray value. The lower edge sampling window image and grayscale image are shown in Fig. 8b and c. The comparison between the prior method and the prior OTSU threshold segmentation effect is shown in Fig. 9. The left ventricular posterior wall contour of a test sample processed by the algorithm in this paper is shown in Fig. 10 below. It can be seen that the detection results different from the common edge detection operators Sobel, Robert, and Prewitt have discontinuities [24] and are relatively broken. The edge obtained by proposed algorithm is complete and continuous, which has the value of analysis and processing.

## Discussion

For the individual, the posterior wall of the left ventricle of the heart cannot be uniform and equal in thickness, and there are differences in thickness at different locations. In this paper, 10 samples of CNN target area images were measured and counted. In the final thickness estimation, after the shortest distance group corresponding to each selected point is counted and the maximum and minimum distance values in the group are removed, the mean value of the remaining distance is taken as the distance between the two edge pixels of the final back wall of the image. The final thickness calculation is carried out by substituting formula 7 and 8. The selection parameters of the lower edge of each sample are as follows: the processing window size is 200 × 200, the starting point intercept is 140, the number of points is 10, and the interval of points is 5.

**Fig. 11:**
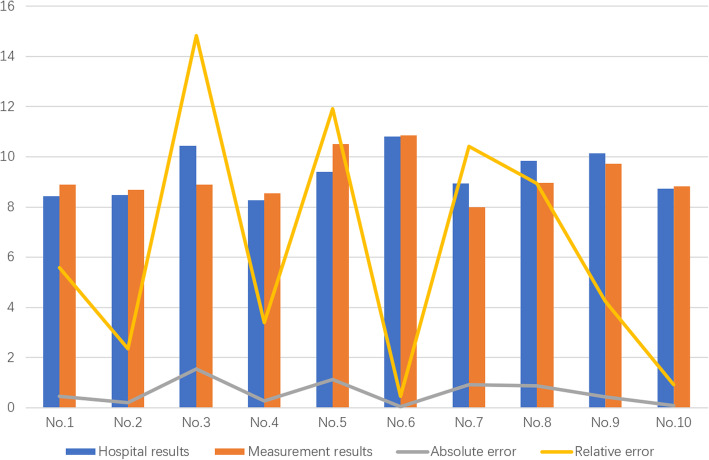
Sample measurement results

The time taken for each echocardiogram sample measurement process, hospital and computer measurement of LVPWT results and error conditions are shown in Table 4, and the statistical graph is shown in Fig. 11. It is considered that the LVPWT measurement given by the hospital is the true thickness of the posterior wall of the left ventricle. It is worth noting that because different doctors have different preferences for selecting points, there is a difference in the LVPWT measured by the same echocardiogram, and even the results of the two measurements taken by the same doctor are different. Clinically, for the echocardiogram of the same patient, the difference (reproducible error) of the measurement results given by different doctors within a reasonable interval is less than 20% (≤2 mm) are considered reasonable and acceptable.

**Table 4:** Statistical measurement results for each sample

Sample no.	Hospital results /mm	Measurement results /mm	Absolute error /mm	Relative error /%	Measurement time /s	Positioning time /s	Total time /s
No.1	8.43	8.90	0.47	5.58	2.46	11.04	13.50
No.2	8.48	8.68	0.20	2.36	2.96	30.02	32.98
No.3	10.45	8.90	1.55	14.83	2.16	26.86	29.02
No.4	8.27	8.55	0.28	3.39	2.13	38.00	40.13
No.5	9.39	10.51	1.12	11.92	2.51	31.64	34.15
No.6	10.80	10.85	0.05	0.46	2.86	20.92	23.78
No.7	8.93	8.00	0.93	10.41	2.80	15.25	18.05
No.8	9.85	8.97	0.88	8.93	2.31	41.03	43.34
No.9	10.15	9.72	0.43	4.24	2.85	52.66	55.51
No.10	8.74	8.82	0.08	0.92	2.18	32.00	34.18

It can be seen from Table 4 that the measurement process in this article takes a short time, and the measurements are completed within 3 s. The sample measurement results in this paper are different from the hospital measurement results and the relative error is less than 15%. The sample test results have been approved by the imaging doctors. Analysis of the measurement process, the possible reasons for the differences in the measurement results are as follows: First, there are subtle differences in the measurement image, the echocardiography of the measurement results given by the hospital is compressed, which is inconvenient for direct measurement. The actual echocardiogram measured in this paper is the valid picture selected after framed echocardiographic video of the same patient. The heart is constantly contracting and relaxing, and the thickness of the posterior wall of the left ventricle changes. The study found that the left ventricular wall systolic thickening rate in men can reach 24.3 to 31.2%, and that in women can reach 24.8 to 30.0%, which is the main reason for the difference. Second, the location of the selected points is different. Doctors usually select only one place for measurement. The thickness of the posterior wall of the left ventricle is uneven, and points were measured at multiple points in this article, which brought differences to the results. Third, in echocardiography, there may be interference of muscle trabeculae attached to posterior wall and false chordae tendineae in interventricular septum. In actual clinical measurement, the doctor will subjectively determine the appropriate measurement position to reduce this interference by observing a period of echocardiography and relying on his own experience. In computer measurement, part of the edge noise in the posterior wall is very close to the posterior wall of the left ventricle, which is difficult to be removed in image enhancement, and will be measured as a part of the posterior wall of the left ventricle, which will affect the measurement results.

## Conclusions

In this paper, using the ability of CNN to autonomously learn image features for classification, a network model was constructed to locate the posterior wall of left ventricle. To solve the over-fitting phenomenon that may occur due to the sample size, this paper performs the Dropout method on the fully connected layer. In order to extract the contours of the posterior wall of the left ventricle in the region located by CNN, this paper uses non-local mean filtering and open operation for image enhancement, and designs an edge detection algorithm based on threshold segmentation for the upper and lower contours. The maximum inter-class variance method adjusts the segmentation threshold twice and completes the contour extraction. The design algorithm was used to select the LVPWT selection point by setting the length threshold to remove a large number of invalid non-target contours and successfully extracting the complete and effective left ventricular posterior wall contour. The analysis results show that the measurement method proposed in this paper has the advantage of less manual intervention. On the premise of preserving the edge details of echocardiography, the image of the target region of the left ventricle was enhanced, and the contour of the posterior wall of the left ventricle was extracted effectively and the thickness was measured. The differences between experimental results and hospital measurement results were compared and analyzed, and the results met the clinical requirements. Many patients with LVH have hypertrophy of other walls such as the septum. The treatment method also has reference significance for parameter measurement of other parts of the heart. Our next work will continue to improve the accuracy and apply the algorithm to other parameters measurement.

## Data Availability

The dataset that support the findings and conclusion of this study are available from the corresponding author on reasonable request. The data are not publicly available due to privacy.

## Abbreviations

LVPWT: Left ventricular posterior wall thickness
LVH: Left ventricular hypertrophy
CNN: Convolutional neural network
NLM: NON-local Mean Filtering
SUSAN: Small Univalue Segment Assimilating Nucleus
